# Meta-analysis of the interaction between serotonin transporter promoter variant, stress, and posttraumatic stress disorder

**DOI:** 10.1038/s41598-017-15168-0

**Published:** 2017-11-28

**Authors:** Mingzhe Zhao, Jiarun Yang, Wenbo Wang, Jingsong Ma, Jian Zhang, Xueyan Zhao, Xiaohui Qiu, Xiuxian Yang, Zhengxue Qiao, Xuejia Song, Lin Wang, Shixiang Jiang, Erying Zhao, Yanjie Yang

**Affiliations:** Psychology Department of the Public Health Institute of Harbin Medical University, No. 157, Baojian Road, Nangang District, Harbin, 150081 China

## Abstract

Exposure to stress predicts the occurrence of posttraumatic stress disorder (PTSD) in individuals harboring the serotonin transporter promoter variant *5-HTTLPR*. We carried out a meta-analysis of studies investigating the interaction between *5-HTTLPR*, stress, and PTSD to clarify the interrelatedness of these factors. We reviewed all relevant studies published in English before May 2016. The Lipták–Stouffer z-score method for meta-analysis was applied to combined data. The z score was separately calculated for the stressful life events, childhood adversity, bi- and triallelic loci, and cross-sectional and longitudinal studies subgroups. A total of 14 studies with 15,883 subjects met our inclusion criteria. We found strong evidence that the presence of *5-HTTLPR* influenced the relationship between stress and PTSD (P = 0.00003), with the strongest effects observed in the cross-sectional and longitudinal groups (P = 0.01 and 2.0 × 10^−6^, respectively). Stressful life events and childhood adversity separately interacted with *5-HTTLPR* in PTSD (P = 2.0 × 10^−8^ and 0.003, respectively). When the studies were stratified by locus classification, the evidence was stronger for the triallelic (P = 4.0 × 10^−8^) than for the biallelic (P = 0.054) locus subgroup. There was strong evidence that *5-HTTLPR* influences the relationship between stress and PTSD.

## Introduction

Posttraumatic stress disorder (PTSD) is a complex and multifactorial anxiety disorder^1^, and the lifetime prevalence estimates 6% worldwide^2^. Since its classification in the third revision of the Diagnostic and Statistical Manual of Mental Disorders (DSM-III), PTSD has increasingly been recognized as a major public health issue^3–5^. In DSM-V, PTSD is characterized by the appearance of three symptom clusters following an acutely traumatic event: re-experiencing (flashbacks and nightmares), avoidance of trauma-related stimuli, and hyperarousal^4^. PTSD differs from other common mental disorders in that exposure to traumatic stressors is a prerequisite for diagnosis.

Stressful circumstances include stressful life events (natural disasters and war) as well as childhood adversity including abuse (e.g., sexual and physical), neglect (failure of caretakers to provide for basic needs such as food), and parental death or divorce can contribute to the etiology of PTSD. Stressful life events are described as circumstances that have a negative impact on individuals that occur close to the onset or relapse of the illness^6^. In the weeks following a stressful life event, most individuals exhibit acute reactions such as negative thoughts or dreams concerning the event, hyper-alertness, irritability, and problems with sleep, memory, and/or concentration^7–11^. It has been shown that clustering of severely stressful life events precedes the occurrence of PTSD or the worsening of symptoms^12^. Childhood adversity is defined as stressful experiences that occur early in life^13^. Numerous studies have demonstrated that childhood adversity is associated with a range of metal disorders including major depression^14^, antisocial disorder, substance abuse^15^, and PTSD^16^. Early life stress can cause permanent changes in brain structure and function^17^, which can in turn influence the response to trauma experienced in adulthood.

PTSD is thought to be influenced not only by environmental but also genetic factors^18,19^; the latter account for at least one third of the variance in PTSD risk^20,21^. Several gene loci associated with PTSD have been identified^18,19,22^, including a polymorphism in the serotonin (5-hydroxytrptamine, 5-HT) transporter (5-HTT) gene (*SLC6A4*) promoter region^18,23^. *SLC6A4* has been reported to modulate sensitivity to stress and susceptibility to psychopathology^24^. *SLC6A4* promoter is modified by elements within the proximal 5 regulatory region known as the *5-HTT* gene-linked polymorphic region (*5-HTTLPR*)^25^, which contains a polymorphism with a rare short (S) and more common long (L) allele; the former is associated with reduced transcriptional efficiency of the promoter^26^, which has been linked to suicidal behavior^27^, depression^28^, and PTSD^29^. *5-HTTLPR* variants have a third functional allele: Lg contains an A > G polymorphism at position 6 of the first two 22-bp imperfect repeats that define the 16-repeat L allele (the single nucleotide polymorphism rs25531)^30^. Thus, *5-HTTLPR* is a triallelic locus from which Lg and S alleles (both reclassified as S’) are expressed at similar levels^13^ and La (reclassified as L’) has higher expression^31,32^.

*SLC6A4* modulates various types of emotional response^33^. Many studies have investigated a potential interaction between *SLC6A4* and the environment in PTSD^29,34–46^, including significant interactions involving both the low-^29,35,37,38,40,44^ and high-^34^ expression genotypes. Some studies reported a significant interaction involving the S allele^43,45^, but this was not confirmed by other investigators^36,39,41,42,46^. These inconsistencies may be due to differences in study design and statistical power.

Two recent meta-analysis assessed the set of studies exploring the association between 5-HTTLPR and PTSD and concluded that evidence did not support the presence of the association^47,48^. However, none of two meta-analyses has assessed the interaction between 5-HTTLRP and stress type in PTSD. In order to clarify the role of SLC6A4 in the etiology of PTSD, we sought to carried out a meta-analysis of published studies on the relationship between 5-HTTLPR, stress, and PTSD. Specifically, three subgroup analysis stratified by type of stressor, study design, and locus classification was first used to detect the interaction effect and sensitivity analysis was conducted to detect publication bias in overall and subgroup analysis. The various studies employed different study designs, making it difficult to merge the results into a single traditional meta-analysis. The Lipták–Stouffer z-score, which is useful in situations where equivalent raw data are not available across relevant studies^49^, has been used to combine P values from many studies of gene and environment interaction^50–52^. Here we used the Lipták–Stouffer z-score to combine information at the level of significance tests to assess whether variations in *5-HTTLPR* influences the relationship between stress and PTSD.

## Materials and Methods

### Studies

In accordance with the Preferred Reporting Items for Systematic Reviews and Meta-Analyses guidelines^53^, we identified candidate studies by examining previous meta-analyses and review articles retrieved from PubMed, Wolters Kluwer, Web of Science, EBSCO, and Elsevier Science Direct from study inception up to May 2016 using the following search terms without restrictions: “posttraumatic stress disorder” or “PTSD”, “serotonin transporter gene” or “*5-HTTLPR*”, “stress”, “trauma”, and “ gene-environment interaction”^47,48^. In addition, we carried out related searches online using Baidu Scholar. We also examined references in prior meta-analyses and review articles to identify eligible publications.

Two investigators independently reviewed the remaining articles to establish eligibility based on predefined inclusion criteria. We included only human studies published in English before May 2016 that investigated the effect of *5-HTTLPR* on the relationship between PTSD and stress, or the relationship between *5-HTTLPR* polymorphisms and stressful events and/or childhood adversity. The genotype distributions in the studies were in Hardy-Weinberg equilibrium. One study was excluded because it used the same data as another study included in the analysis^54^. A total of 14 independent investigations with 15,883 study subjects met the inclusion criteria.

To ascertain whether results were affected by study design characteristics, we analyzed subgroups based on three variables, some of which have been defined in recent review articles^51,52^. We stratified studies by type of stressor (stressful life events and childhood adversity), study design (cross-sectional and longitudinal), and by locus classification (bi- and triallelic loci).

### Quality assessment

The methodological quality of eligible studies was evaluated according to a quality checklist derived from Strengthening the Reporting of Observational Studies in Epidemiology checklists^55,56^, which have been used in some gene-environment interaction meta-analyses^50–52^. In accordance with current guidelines and prior studies^50–52^, included studies were not weighted by quality scores or excluded based on low scores. We nonetheless describe data quality in Supplementary Materials of Table S1 for readers to evaluate.

### P value extraction

Two authors independently extracted P values from each included study without divergence. If a study did not report an exact statistical outcome (e.g., the article stated only P > 0.05), the authors were contacted to obtain more precise values. If that was unsuccessful, a P value of 1 (indicating a lack of outcome) was assigned. In some instances, several P values were reported due to differences in PTSD scales or sample subsets among studies. Therefore, weighted mean P values were used in our analyses. If reported analyses corresponded to different groups, the mean of P values for each group were incorporated into the overall analysis.

### Statistical analysis

After combining eligible studies, we applied the Lipták–Stouffer z-score to obtain an aggregate value based on the significance level of tests weighed by sample size. We first converted extracted P values to one-tailed metrics where P values Z<0.50 indicated greater sensitivity to S/Lg allele stress and those >0.50 corresponded to greater sensitivity to L/La stress in PTSD. We then converted these P values to z scores, with positive and negative z scores corresponding to P values less and greater than 0.05, respectively. The z scores were incorporated into the following formula:

$${Z}_{w}=\frac{{\sum }_{i=1}^{k}{W}_{i}{Z}_{i}}{\sqrt{{\sum }_{i=1}^{k}{W}_{i}^{2}}}$$

where the weighting factor W_i_ corresponds to the study sample size; Z_i_ is the study z score; and k is the total number of studies. Z_w_ conformed to a normal distribution and the corresponding probability was obtained from a standard normal distribution table. This statistical procedure was applied to all studies and to the stratified analyses.

To determine whether any single study had a disproportionate influence on our results, we carried out sensitivity analyses by computing Z_w_ after removing each study in turn. To gauge publication bias, we calculated the fail-safe N for the overall analysis and for each stratified analysis, since unreported studies could potentially influence the conclusions of our meta-analysis. Consistent with previous reports^50,51^, we counted the number of studies with an assigned P value of 0.50 and the average sample size of studies incorporated into the weighted Lipták–Stouffer analysis that yielded a non-significant result. The ratio of fail-safe N to the number of published studies provided an estimate of publication bias in our results.

### Date availability statement

The authors declare that the data in this research is available.

## Results

### Literature search results

**Figure 1:**
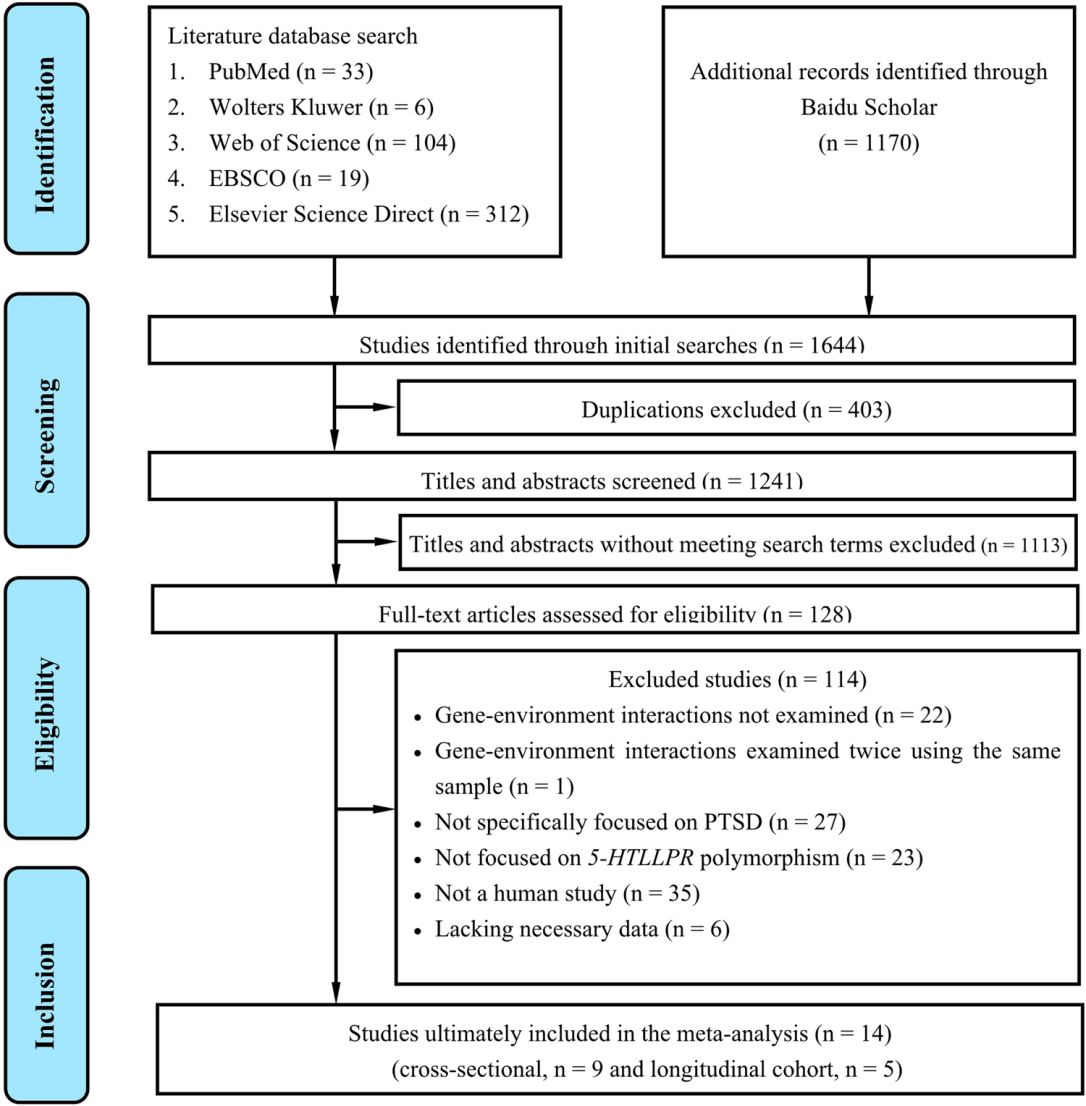
Flow chart of study screening process.

The study selection procedure is shown in Fig. 1. We identified 1644 potentially relevant records through literature searches; 403 were duplicated articles and were excluded. After screening titles and abstracts, 1113 additional articles were excluded, leaving 128 full-text articles for eligibility assessment. We ultimately included 14 studies in our meta-analysis that fulfilled all inclusion criteria. The characteristics of these studies are shown in Table 1 and Supplementary Materials of Table S2.

**Table 1:** Studies on the interaction between *5-HTTLPR* polymorphism, life stress, and PTSD included in the meta-analysis.

Study	No. of participants	Males, N (%)	Age, range or mean (years)	Ancestry	Study design	Stressor	Stressor measure	Diagnostic instrument	PTSD measure	Allele sensitivity	Averaged 1-tailed P value	Lipták-Stouffer P value after study exclusion
Kilpatrick *et al*., 2007	589	374 (63.5)	≥ 60 (76.6%)	Mixed	Cross-sectional	SLEs	MOSM	DSM-IV	Last 6–9 months	S/Lg	0.047	5.0 × 10^−5^
Grabe *et al*., 2009	3045	1571 (51.6)	20–79	European	Longitudinal	CA& SLEs	SCID	SCID	lifetime	La	0.005	6.5 × 10^−4^
Xie *et al*., 2009	1252	651 (52)	17–79	Mixed	Cross-sectional	CA& SLEs	SSADDA	SSADDA	lifetime	S/Lg	0.019	1.1 × 10^−4^
Kolassa *et al*., 2009	408	218 (53.4)	17–68	African	Cross-sectional	SLEs	PDS	DSM-IV	lifetime	S	0.055	4.0 × 10^−5^
Holman *et al*., 2011	711	Not reported	No report	European	Longitudinal	SLEs	DIS trauma section	PCL	Last 1 year	S/Lg	0.005	8.0 × 10^−5^
Mercer *et al*., 2012	1045	0	18–45	Mixed	Longitudinal	SLEs	TLEQ/DEQ	TLEQ/DEQ	Last 30 days	S/Lg	0.003	1.3 × 10^−4^
Xie *et al*., 2012	6430	3551 (55)	17–85	Mixed	Case-control	CA	Self-edited questionnaire	SSADDA	lifetime	S	0.122	0.00000
Wald *et al*., 2013	1085	1085 (100)	18–24	Mixed	Longitudinal	SLEs	Self-edited questionnaire	PCL	Current	S/Lg	0.003	1.4 × 10^−4^
Pietrzak *et al*., 2013	149	63 (41.1)	18–92	Mixed	Cross-sectional	SLEs	Self-edited questionnaire	PCL	Last 2–5 months	S	0.096	3.0 × 10^−5^
La Greca *et al*., 2013	116	53 (46)	8.85	Mixed	Cross-sectional	SLEs	HURTE-R	PTSD-RI-R	After 8 months	S	0.500	3.0 × 10^−5^
Walsh *et al*., 2014	682	254 (37)	43.63	African	Cross-sectional	CA	CTQ	PCL-S	lifetime	S	0.009	7.0 × 10^−5^
Telch *et al*., 2015	133	114 (85.7)	23.5	Mixed	Longitudinal	SLEs	CEL	PCL-S	Current	S/Lg	0.012	4.0 × 10^−5^
Tian *et al*., 2015	183	99 (54.1)	15.2	Asian	Cross-sectional	SLEs	Self-edited questionnaire	PCL-C	Last 3 years	S	0.008	4.0 × 10^−5^
Drevo *et al*., 2016	55	0	18–70	Mixed	Cross-sectional	SLEs	Self-edited questionnaire	PSS-SR-17	Current	S	0.500	3.0 × 10^−5^
**Total:**	**15883**										**0.00003**	
**Average:**	**1135**											

CA, childhood adversity; DSM-IV, Diagnostic and Statistical Manual of Mental Disorders, fourth revision; SLEs, stressful life events; SCID, the Structured Clinical Interview for DSM-IV; SSADDA, Semi-Structured Assessment for Drug Dependence and Alcoholism interview; PCL, the PTSD Checklist-Civilian Version; TLEQ, Traumatic Life Events Questionnaire; DEQ, the Distressing Event Questionnaire; PTSD-RI-R, the Posttraumatic Stress Disorder-Reaction Index for Children-Revised; PCL-S, the PTSD Checklist-Specific version; PCL-C, the PTSD Checklist-Civilian Version; PSS-SR-17, Posttraumatic Stress Disorder Symptom Scale-Self Report-17; MOSM, Medical Outcome Study Module; PDS, Posttraumatic Diagnostic Scale; DIS, Diagnostic Interview Schedule; HURTE-R, The Hurricane Related Traumatic Experiences-Revised; CTQ, The Childhood Trauma Questionnaire; CEL, Combat Experiences Log.

### Overall meta-analysis

**Figure 2:**
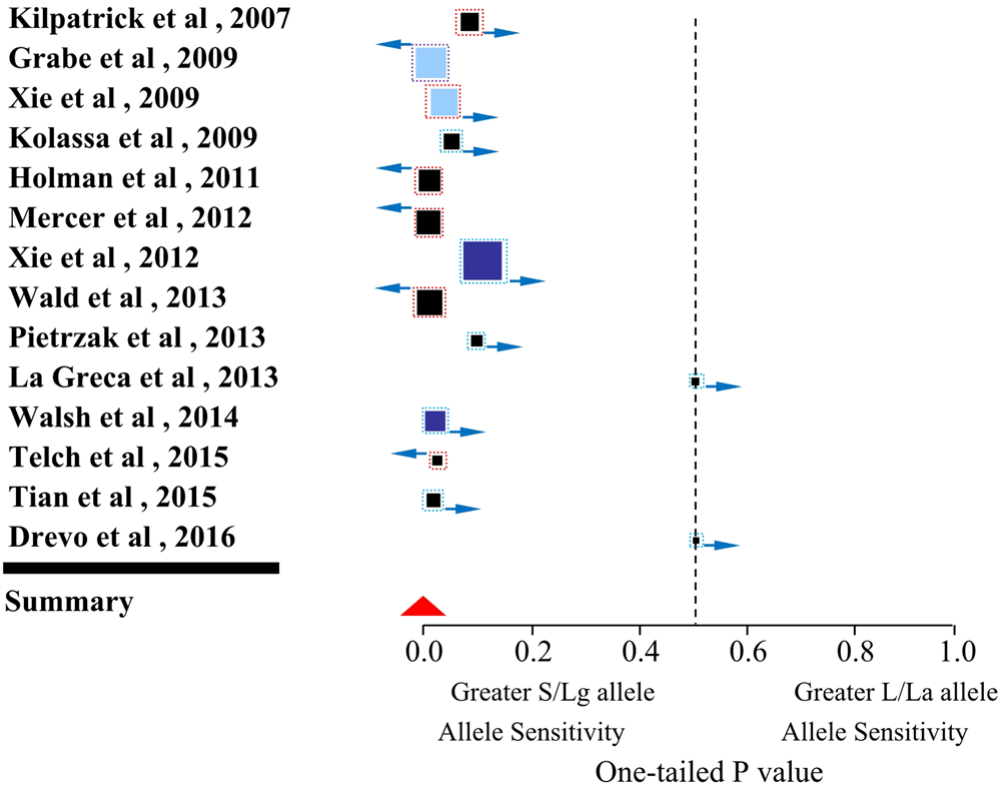
Forest plot of 14 human observational studies for the interaction of 5-HTTLPR genotype and life stress on PTSD. The squares mark indicate the one-tailed P value for each study, where lower values denote greater stress sensitivity of S allele carriers and higher values correspond to greater stress sensitivity of L allele carriers. The size of the box reflects relative sample size. The red triangle indicates the overall result of meta-analysis. Black squares mark studies that indexed stressful life events; Dark blue indicates childhood adversity; and light blue indicates studies that included both stressful life events and childhood adversities; The squares with red border indicate S/Lg alleles; The squares with blue border indicate S alleles; and the squares with purple border indicate La alleles. The squares with w-resize indicate longitudinal studies and the squares with e-resize indicate cross-sectional studies.

The 14 studies (with a total of 15,883 study subjects) were pooled to assess the interaction between *5-HTTLPR* polymorphism, stress, and PTSD. We found strong evidence that *5-HTTLPR* influences the relationship between stress and PTSD, with the S allele associated with an increased risk of developing PTSD under stress (P = 3.0 × 10^−5^) (Fig. 2). The results remained robust when each study was removed in turn from the overall analysis—that is, the overall P values remained significant (3.0 × 10^−5^ < P < 0.0001) (Table 1).

With respect to publication bias, a non-significant result (P = 0.05) in the overall analysis would require more than 226 unpublished or undiscovered studies with an average sample size of n = 1135 and non-significant results (P = 0.50), corresponding to a fail-safe ratio of 16 studies excluded from the meta-analysis for every included study.

Previous studies provided evidence for distinct interaction effects of child adversity and stressful life events with *5-HTTLPR* ^50^ and *brain-derived neurotrophic factor* ^51^ in depression; with respect to studies included in the present meta-analysis, results pertaining to the impact of these stressors and the *5-HTTLPR* gene in PTSD were examined.

### Childhood adversity

Four studies were pooled for a total of 11,409 study subjects; the combined results revealed a significant interaction between *5-HTTLPR* and childhood adversity (P = 0.003) (Fig. 2). The sensitivity analysis showed that the results remained significant after each study was removed in turn (1 × 10^−4^ < P < 0.03) (Table 2). Obtaining a significant result (P = 0.05) would require more than 21 unpublished or undiscovered studies with an average sample size of n = 2852 and non-significant results (P = 0.50). This yields a fail-safe ratio of five excluded studies for every study included in the present meta-analysis.

**Table 2:** Studies included in the life stress group meta-analysis.

Source, year	No. of subjects	1-Tailed P value	P value after study exclusion
Kilpatrick *et al*., 2007	589	0.047	6.02 × 10^−8^
Grabe *et al*., 2009	3045	0.005	3.70 × 10^−9^
Xie *et al*., 2009	1252	0.019	1.80 × 10^−7^
Kolassa *et al*., 2009	408	0.055	4.44 × 10^−8^
Holman *et al*., 2011	711	0.005	1.74 × 10^−7^
Mercer *et al*., 2012	1045	0.003	4.31 × 10^−7^
Wald *et al*., 2013	1085	0.003	4.64 × 10^−7^
Pietrzak *et al*., 2013	149	0.096	2.63 × 10^−8^
La Greca *et al*., 2013	116	0.500	1.98 × 10^−8^
Telch *et al*., 2015	133	0.012	3.09 × 10^−8^
Tian *et al*., 2015	183	0.008	3.74 × 10^−7^
Drevo *et al*., 2016	55	0.500	2.01 × 10^−8^
**Total:**	**8771**		
**Average sample size:**	**731**	**2.0 × 10** ^**−8**^	

### Stressful life events

Twelve studies were pooled for a total of 8771 study subjects; the results revealed a significant interaction between stressful life events and *5-HTTLPR* polymorphism (P = 2.0 × 10^−8^) (Fig. 2). The sensitivity analysis showed that the P value remained significant after removing each study in turn and calculating the outcome (3.0 × 10^−9^ < P < 4.0 × 10^−7^) (Table 3). More than 166 unpublished or undiscovered studies with a null effect (P = 0.50) and average sample size of n = 731 would be required for this to be a non-significant outcome in the stratified analysis (P = 0.05), corresponding to a fail-safe ratio of 14 excluded studies for every study included in this meta-analysis.

**Table 3:** Studies included in the childhood adversity group meta-analysis.

Source, year	No. of Participants	1-Tailed P value	P Value after study exclusion
Grabe *et al*., 2009	3045	0.005	0.0378
Xie *et al*., 2009	1252	0.019	0.0088
Xie *et al*., 2012	6430	0.122	0.0001
Walsh *et al*., 2014	682	0.009	0.0065
**Total:**	**11409**		
**Average sample size:**	**2852**	**0.0035**	

The time at which a stressor is measured can affect the gene and environment interaction effect^57^, while recalling adversity over long periods of time may increase the risk of forgetting or discounting events^24^. This type of bias can largely be avoided in longitudinal studies. We carried out a subgroup analysis based on study design to determine whether outcomes differed between cross-sectional and longitudinal studies.

### Cross-sectional studies

We identified nine studies with a cross-sectional study design (9864 subjects) for which results were available in separate cross-sectional studies. The meta-analysis revealed a significant interaction between *5-HTTLPR* and stress (P = 0.01) (Fig. 2), and the sensitivity analysis revealed that this effect persisted after each study was removed in turn (3.0 × 10^−5^ < P < 0.04) (Table 4). More than 49 unpublished or undiscovered studies with a sample size of n = 1096 and a non-significant result (P = 0.50) would be required for the outcomes in the stratified analysis to be non-significant (P = 0.05). This yields a fail-safe ratio of five studies excluded for every study included in the meta-analysis.

**Table 4:** Studies included in the cross-sectional group meta-analysis.

Source, year	No. of participants	1-Tailed P value	P Value after study exclusion
Kilpatrick *et al*., 2007	589	0.047	0.024
Xie *et al*., 2009	1252	0.019	0.040
Kolassa *et al*., 2009	408	0.055	0.022
Xie *et al*., 2012	6430	0.122	3.0 × 10^−5^
Pietrzak *et al*., 2013	149	0.096	0.018
La Greca *et al*., 2013	116	0.500	0.017
Walsh *et al*., 2014	682	0.009	0.030
Tian *et al*., 2015	183	0.008	0.020
Drevo *et al*., 2016	55	0.500	0.017
**Total:**	**9864**		
**Average sample size:**	**1096**	**0.017**	

### Longitudinal studies

Five studies were pooled with a total of 6019 study subjects, revealing a significant interaction between *5-HTTLPR* and life stress (P = 2.0 × 10^−6^) (Fig. 2). The sensitivity analysis suggested that the results were still significant after removing each study in turn (8.0 × 10^−7^ < P < 9.0 × 10^−5^) (Table 5). For these results to be non-significant (P = 0.05), more than 56 unpublished analyses or undiscovered studies with an average sample size of n = 1203 and non-significant results (P = 0.50) would be required, yielding a fail-safe ratio of 11 excluded studies for every study included in the meta-analysis.

**Table 5:** Studies included in the longitudinal group meta-analysis.

Source, year	No. of participants	1-Tailed P value	P value after study exclusion
Grabe *et al*., 2009	3045	0.005	9.0 × 10^−7^
Holman *et al*., 2011	711	0.005	2.0 × 10^−5^
Mercer *et al*., 2012	1045	0.003	9.0 × 10^−5^
Wald *et al*., 2013	1085	0.003	4.0 × 10^−5^
Telch *et al*., 2015	133	0.012	3.0 × 10^−6^
**Total:**	**6019**		
**Average sample size:**	**1204**	**2.0 × 10** ^**−6**^	

### Biallelic locus

We conducted a subgroup analysis stratified by locus to explore the interaction between *5-HTTLPR* polymorphism, life stress, and PTSD. Seven studies were pooled with a total of 8023 study subjects; the results revealed a trend towards a significant interaction between a biallelic *5-HTTLPR* locus, life stress, and PTSD (P = 0.054) (Fig. 2). The sensitivity analysis indicated that the results remained non-significant after removing each study in turn (0.054 < P < 0.086), with the exception of one study^35^ (Table 6).

**Table 6:** Studies included in the S/Lg allele group meta-analysis.

Source, year	No. of participants	1-tailed P value	P value after study exclusion
Kilpatrick *et al*., 2007	589	0.047	2.0 × 10^−7^
Xie *et al*., 2009	1252	0.019	2.0 × 10^−7^
Holman *et al*., 2011	711	0.005	1.0 × 10^−6^
Mercer *et al*., 2012	1045	0.003	2.0 × 10^−6^
Wald *et al*., 2013	1085	0.003	2.0 × 10^−6^
Telch *et al*., 2015	133	0.012	9.0 × 10^−8^
**Total:**	**4815**		
**Average sample size:**	**803**	**4.0 × 10** ^**−8**^	

### Triallelic locus

Six studies were pooled with a total of 4815 subjects to assess the interaction between a triallelic *5-HTTLPR* locus, life stress, and PTSD; a significant interaction was observed (P = 4.0 × 10^8^) (Fig. 2). The results remained significant in the sensitivity analysis when each study was removed in turn from the analysis (2.0 × 10^−7^ < P < 2.0 × 10^−6^) (Table 7). To render the outcomes non-significant (P = 0.05), more than 67 unpublished or undiscovered studies with a sample size of n = 802 and non-significant results (P = 0.50) would be required. This yielded a fail-safe ratio of 11 excluded studies for every study included in the meta-analysis.

**Table 7:** Studies included in the S allele group meta-analysis.

Source, year	No. of participants	1-tailed P value	P value after study exclusion
Kolassa *et al*., 2009	408	0.055	0.06
Xie *et al*., 2012	6430	0.122	2.0 × 10^−4^
Pietrzak *et al*., 2013	149	0.096	0.05
La Greca *et al*., 2013	116	0.500	0.05
Walsh *et al*., 2014	682	0.009	0.08
Tian *et al*., 2015	183	0.008	0.06
Drevo *et al*., 2016	55	0.500	0.05
**Total:**	**8023**		
**Average sample size:**	**1146**	**0.054**	

## Discussion

This is the first meta-analysis investigating the interaction between *5-HTTLPR* polymorphism, stress, and PTSD. We found that *5-HTTLPR* polymorphism influenced the relationship between stress and PTSD, with the less frequent S allele associated with increased stress sensitivity. When the meta-analysis was stratified by type of stressor, we found that stressful life events and childhood adversity independently interacted with *5-HTTLPR* in PTSD; when the analysis was stratified by study design, we found interactions between *5-HTTLPR* and stress in both cross-sectional and longitudinal groups; and when the analysis was stratified by allele classification, the results were more robust for the triallelic models group while the interaction effect for biallelic models failed to reach the predetermined level of significance.

The overall results are consistent with a recent qualitative review^58^, and are in accordance with studies that have reported increased stress reactivity among *5-HTTLRP* S allele carriers^59,60^. Animal studies have also demonstrated that functional variations in the *5-HTT* gene affect behavioral response to stress. Specifically, *5-HTT* knockout mice showed increased hypothalamic-pituitary-adrenal axis activation in response to both physical and psychological stressors^61,62^, as well as defects in cortical development and altered expression of 5-HT receptor subtypes^63–65^. Moreover, evidence from studies in mice and non-human primates have shown that *5-HTT* gene variants are associated with changes in central nervous system biochemistry and behaviors linked to stress sensitivity^66,67^.

Of the 14 studies that investigated the influence of stressful life events and childhood adversity on PTSD, 12 examined the interaction effects between stressful life events and *5-HTTLPR*; eight of these reported a significant interaction for PTSD^29,34,35,37,38,40,44,45^, whereas four did not find any evidence of interaction^36,41,42,46^. Four of the 14 studies investigated the interaction between childhood adversity and *5-HTTLPR*, with three reporting a significant interaction for PTSD^35,42,43^ and one finding no supporting evidence^39^. The meta-analysis revealed significant interaction effects for stressful life events and childhood adversity separately interacting with *5-HTTLPR* in PTSD. Our results are consistent with studies that have reported an association between *5-HTTLPR* and neural responses to traumatic reminders and cognitive control of emotions in PTSD patients and the persistent effects of stressful life events and childhood adversity on hippocampal volume^68–71^.

Nine of the 14 studies used a cross-sectional design^29,35,36,39,41–43,45,46^, with four reaching a conventional significance level^29,35,43,45^; meanwhile, significance was attained by all five studies that used a longitudinal design^34,37,38,40,44^. The meta-analysis of both cross-sectional and longitudinal studies showed that *5-HTTLPR* interacted with stress and PTSD. At least one study has failed to detect a gene–environment interaction between *5-HTTLPR* polymorphism and life events in the months immediately preceding PTSD onset^72^. However, most studies have measured life events in the 5 years prior to PTSD. Retrospective recall of adversity is associated with increased risk of forgetting or discounting events^24^; thus, when only a lifetime diagnosis of PTSD is available, information about the relative timing of stressors and PTSD is lost. This bias can be avoided to a greater extent in longitudinal than in cross-sectional studies.

We evaluated the association between loci using bi- and triallelic models to determine whether the latter better reflects the interaction between the *5-HTTLPR* polymorphism, stress, and PTSD. We found evidence supporting the interaction in the triallelic but not in the biallelic group. Our approach was based on the reclassification of alleles according to lower and higher levels of expression, which is more precise for exploring the interaction between *5-HTTLPR* polymorphism, stress, and PTSD^30,32^.

There were some limitations to the present meta-analysis. Firstly, several of the included studies may have limited power due to their small sample size^41,42,44,46^. Secondly, since we combined studies at the level of P values, the quality of primary studies may have affected our results. Some primary studies conducted separate tests on different sample subgroups or multiple PTSD measures; we guarded against false-positive results resulting from this potential bias by using an average of reported P values. Finally, we could not estimate the magnitude of genetic effect and how it compares to the interaction effect size^73^.

In conclusion, we found that 5-HTTLPR influences the relationship between stress and PTSD. Further studies which focus on Gene × Environment interaction are needed to better understand the role of this polymorphism in PTSD risk. Our analysis identified study characteristics that could potentially affect study results such as type of stressor, study design, and allele classification. Childhood adversity and stressful life events could be two good candidate environmental risk factors in G × E research. The triallelic models approach (S/La/Lg) altered the results of meta-analysis comparing with the biallelic models approach(S/L). Special attention should be paid to the triallelic polymorphism in the relationship between 5-HTTLPR, stress and PTSD. These findings provide a basis for designing more rigorous studies on gene–environment interactions in PTSD in the future.

## Electronic supplementary material

Supplementary Materials

## References

[1] Xie P (2009). Interactive effect of stressful life events and the serotonin transporter 5-HTTLPR genotype on posttraumatic stress disorder diagnosis in 2 independent populations. Arch Gen Psychiatry.

[2] Yehuda, R. *et al*. Post-traumatic stress disorder. *Nature Reviews Disease Primers*, 15057, 10.1038/nrdp.2015.57 (2015). 10.1038/nrdp.2015.57 27189040

[3] Pichot P (1986). DSM-III: the3d edition of the Diagnostic and Statistical Manual of Mental Disorders from the American Psychiatric Association. Revue neurologique.

[4] Yehuda R (2015). Post-traumatic stress disorder. *Nature reviews*. Disease primers.

[5] Kessler, R. C. Posttraumatic stress disorder: the burden to the individual and to society. *The Journal of clinical psychiatr*y **61 Suppl** 5, 4–12; discussion13–14 (2000). 10761674

[6] Ezquiaga E, Gutierrez JLA, López AG (1987). Psychosocial factors and episode number in depression. Journal of Affective Disorders.

[7] Bryant RA, Friedman MJ, Spiegel D, Ursano R, Strain J (2011). A review of acute stress disorder in DSM-5. Depression and anxiety.

[8] Frommberger UH (1998). Prediction of posttraumatic stress disorder by immediate reactions to trauma: a prospective study in road traffic accident victims. European archives of psychiatry and clinical neuroscience.

[9] Isserlin L, Zerach G, Solomon Z (2008). Acute stress responses: A review and synthesis of ASD, ASR, and CSR. The American journal of orthopsychiatry.

[10] Rothbaum BO, Foa EB, Riggs DS, Murdock T, Walsh W (1992). A prospective examination of post-traumatic stress disorder in rape victims. Journal of Traumatic Stress.

[11] Sloan P (1988). Post-traumatic stress in survivors of an airplane crash-landing: A clinical and exploratory research intervention. Journal of Traumatic Stress.

[12] Friedman, M. J., Resick, P. A. & Keane, T. M. PTSD: Twenty-five years of progress and challenges (2007).

[13] Hu X (2005). *An expanded evaluation of the relationship of* four alleles to the level of response to alcohol and the alcoholism risk. Alcoholism Clinical & Experimental Research.

[14] Widom CS, DuMont K, Fau J, Czaja SJ (2007). A prospective investigation of major depressive disorder and comorbidity in abused and neglected children grown up. Arch Gen Psychiatry.

[15] Kendler KS (2000). Childhood sexual abuse and adult psychiatric and substance use disorders in women: an epidemiological and cotwin control analysis. Arch Gen Psychiatry.

[16] Copeland WE, Keeler G, Angold A, Costello EJ (2007). Traumatic events and posttraumatic stress in childhood. Archives of General Psychiatry.

[17] Teicher MH (2003). The neurobiological consequences of early stress and childhood maltreatment. Neuroscience & Biobehavioral Reviews.

[18] Broekman BFP, Olff M, Boer F (2007). The genetic background to PTSD. Neuroscience & Biobehavioral Reviews.

[19] Koenen KC (2007). Genetics of posttraumatic stress disorder: Review and recommendations for future studies. Journal of Traumatic Stress.

[20] Stein MB, Jang KL, Taylor S, Vernon PA, Livesley WJ (2002). Genetic and environmental influences on trauma exposure and posttraumatic stress disorder symptoms: a twin study. Am J Psychiatry.

[21] True WR (1993). A twin study of genetic and environmental contributions to liability for posttraumatic stress symptoms. Archives of General Psychiatry.

[22] de Quervain DJ (2007). A deletion variant of the alpha2b-adrenoceptor is related to emotional memory in Europeans and Africans. Nature Neuroscience.

[23] Lee HJ (2005). Influence of the serotonin transporter promoter gene polymorphism on susceptibility to posttraumatic stress disorder. Depression and Anxiety.

[24] Caspi A, Hariri AR, Holmes A, Uher R, Moffitt TE (2010). Genetic sensitivity to the environment: the case of the serotonin transporter gene and its implications for studying complex diseases and traits. American Journal of Psychiatry.

[25] Caspi A (2003). Influence of Life Stress on Depression: Moderation by a Polymorphism in the 5-HTT Gene. Science.

[26] Lesch KP (1996). Association of Anxiety-Related Traits with a Polymorphism in the Serotonin Transporter Gene Regulatory Region. Science.

[27] Li, D. & He, L. *Meta-analysis supports association between serotonin transporter (5-HTT) and suicidal behavior*. (Willard Grant Press, 2007). 10.1038/sj.mp.4001890 16969368

[28] Lotrich FE, Pollock BG (2004). Meta-analysis of serotonin transporter polymorphisms and affective disorders. Psychiatric Genetics.

[29] Kilpatrick DG (2007). The serotonin transporter genotype and social support and moderation of posttraumatic stress disorder and depression in hurricane-exposed adults. American Journal of Psychiatry.

[30] Nakamura M, Ueno S, Sano A, Tanabe H (2000). The human serotonin transporter gene linked polymorphism (5-HTTLPR) shows ten novel allelic variants. Molecular Psychiatry.

[31] Zalsman G (2006). Association of a Triallelic Serotonin Transporter Gene Promoter Region (5-HTTLPR) Polymorphism With Stressful Life Events and Severity of Depression. American Journal of Psychiatry.

[32] Parsey RV (2006). Effect of a triallelic functional polymorphism of the serotonin-transporter-linked promoter region on expression of serotonin transporter in the human brain. American Journal of Psychiatry.

[33] Caspi A, Moffitt TE (2006). Gene-environment interactions in psychiatry: joining forces with neuroscience. Nature Reviews Neuroscience.

[34] Grabe HJ (2009). Serotonin transporter gene (SLC6A4) promoter polymorphisms and the susceptibility to posttraumatic stress disorder in the general population. American Journal of Psychiatry.

[35] Xie P (2009). Interactive effect of stressful life events and the serotonin transporter 5-HTTLPR genotype on posttraumatic stress disorder diagnosis in 2 independent populations. Archives of general psychiatry.

[36] Kolassa IT (2010). Association study of trauma load and SLC6A4 promoter polymorphism in posttraumatic stress disorder: evidence from survivors of the Rwandan genocide. Journal of Clinical Psychiatry.

[37] Holman EA, Lucas-Thompson RG, Lu T (2011). Social constraints, genetic vulnerability, and mental health following collective stress † ‡. Journal of Traumatic Stress.

[38] Mercer KB (2011). Acute and posttraumatic stress symptoms in a prospective gene x environment study of a university campus shooting. Archives of General Psychiatry.

[39] Xie, P., Kranzler, H. R., Farrer, L. & Gelernter, J. *Serotonin transporter 5-HTTLPR genotype moderates the effects of childhood adversity on posttraumatic stress disorder risk: A replication study*. (G. Narr, 2012). 10.1002/ajmg.b.32068 PMC3428016 22693124

[40] Wald, I. *et al*. Attention to threats and combat-related posttraumatic stress symptoms: prospective associations and moderation by the serotonin transporter gene. **70**, 1–9 (2013). 10.1001/2013.jamapsychiatry.188 PMC4469781 23407816

[41] Pietrzak RH, Galea S, Southwick SM, Gelernter J (2013). Examining the relation between the serotonin transporter 5-HTTPLR genotype x trauma exposure interaction on a contemporary phenotypic model of posttraumatic stress symptomatology: a pilot study. Journal of Affective Disorders.

[42] Lai BS, La Greca AM, Auslander BA, Short MB (2013). Children’s risk and resilience following a natural disaster: genetic vulnerability, posttraumatic stress, and depression. Journal of Affective Disorders.

[43] Walsh K, Uddin M, Soliven R, Wildman DE, Bradleydavino B (2014). Associations between the SS Variant of 5-HTTLPR and PTSD Among Adults with Histories of Childhood Emotional Abuse: Results from Two African American Independent Samples. Journal of Affective Disorders.

[44] Telch, M. J. *et al*. 5-HTTLPR genotype potentiates the effects of war zone stressors on the emergence of PTSD, depressive and anxiety symptoms in soldiers deployed to iraq. *World Psychiatry Official Journal of the World Psychiatric Association* **14**, in press (2015). 10.1002/wps.20215 PMC4471977 26043338

[45] Tian Y (2015). Association of Genetic Factors and Gene-Environment Interactions With Risk of Developing Posttraumatic Stress Disorder in a Case-Control Study. Biological Research for Nursing.

[46] Drevo, S. *et al*. The Role of Social Environment and Gene Interactions on Development of Posttraumatic Stress Disorder (2016).

[47] Gressier F (2013). The 5-HTTLPR Polymorphism and Posttraumatic Stress Disorder: A Meta-Analysis. Journal of Traumatic Stress.

[48] Navarromateu F, Escámez T, Koenen KC, Alonso J, Sánchezmeca J (2013). Meta-analyses of the 5-HTTLPR polymorphisms and post-traumatic stress disorder. Plos One.

[49] Hedges LV, Olkin I (1986). Statistical Methods for Meta-Analysis. American Journal of Sociology.

[50] Karg K, Burmeister M, Shedden K, Sen S (2011). The Serotonin Transporter Promoter Variant (5-HTTLPR), Stress, and Depression Meta-Analysis Revisited: Evidence of Genetic Moderation. Archives of General Psychiatry.

[51] Hosang GM (2014). Interaction between stress and the BDNF Val66Met polymorphism in depression: a systematic review and meta-analysis. BMC Medicine.

[52] Byrd AL, Manuck SB (2014). MAOA, childhood maltreatment, and antisocial behavior: meta-analysis of a gene-environment interaction. Biological Psychiatry.

[53] Moher D, Liberati A, Tetzlaff J, Altman DG, Group P (2009). Preferred reporting items for systematic reviews and meta-analyses: the PRISMA statement. PLoS Med.

[54] Koenen KC (2009). Modification of the association between serotonin transporter genotype and risk of posttraumatic stress disorder in adults by county-level social environment. American Journal of Epidemiology.

[55] Little J, Birkett N (2009). STrengthening the REporting of genetic association studies (STREGA)- An extension of the STROBE statement. Genetic Epidemiology.

[56] Elm EV (2007). Strengthening the reporting of observational studies in epidemiology (STROBE) statement: guidelines for reporting observational studies. Der Internist.

[57] Brown GW, Harris TO (2008). Depression and the serotonin transporter 5-HTTLPR polymorphism: A review and a hypothesis concerning gene-environment interaction. Journal of Affective Disorders.

[58] Koenen KC, Amstadter Ab Fau - Nugent NR, Nugent NR (2009). Gene-environment interaction in posttraumatic stress disorder: an update. J Trauma Stress.

[59] Heinz A (2007). Serotonin transporter genotype (5-HTTLPR): effects of neutral and undefined conditions on amygdala activation. Biological Psychiatry.

[60] Munafò MR, Brown SM, Hariri AR (2008). Serotonin transporter (5-HTTLPR) genotype and amygdala activation: a meta-analysis. Biological Psychiatry.

[61] Li Q (2006). Cellular and molecular alterations in mice with deficient and reduced serotonin transporters. Molecular Neurobiology.

[62] Li Q (1999). Reduction of 5-hydroxytryptamine (5-HT)(1A)-mediated temperature and neuroendocrine responses and 5-HT(1A) binding sites in 5-HT transporter knockout mice. The Journal of pharmacology and experimental therapeutics.

[63] Mössner R (2004). Quantitation of 5HT3 receptors in forebrain of serotonin transporter deficient mice. Journal of Neural Transmission.

[64] Fabre V (2000). Altered expression and functions of serotonin 5-HT 1A and 5-HT 1B receptors in knock-out mice lacking the 5-HT transporter. European Journal of Neuroscience.

[65] Persico AM (2001). Barrel pattern formation requires serotonin uptake by thalamocortical afferents, and not vesicular monoamine release. Journal of Neuroscience the Official Journal of the Society for Neuroscience.

[66] Barr CS (2004). Sexual Dichotomy of an Interaction between Early Adversity and the Serotonin Transporter Gene Promoter Variant in Rhesus Macaques. Proceedings of the National Academy of Sciences of the United States of America.

[67] Carneiro AMD (2009). Functional Coding Variation in Recombinant Inbred Mouse Lines Reveals Multiple Serotonin Transporter-Associated Phenotypes. Proceedings of the National Academy of Sciences of the United States of America.

[68] Hauser MA (2011). Serotonin transporter gene polymorphisms and brain function during emotional distraction from cognitive processing in posttraumatic stress disorder. BMC Psychiatry.

[69] Rabl U (2014). Additive Gene–Environment Effects on Hippocampal Structure in Healthy Humans. Journal of Neuroscience the Official Journal of the Society for Neuroscience.

[70] Teicher MH, Anderson CM, Polcari A (2012). Childhood maltreatment is associated with reduced volume in the hippocampal subfields CA3, dentate gyrus, and subiculum. Proceedings of the National Academy of Sciences.

[71] Gray JD, Rubin TG, Hunter RG, Mcewen BS (2013). Hippocampal gene expression changes underlying stress sensitization and recovery. Molecular Psychiatry.

[72] Brown G, Harris T (2008). Depression and the serotonin transporter 5-HTTLPR polymorphism: a review and a hypothesis concerning gene-environment interaction. Journal of Affective Disorders.

[73] Clarke H (2010). Association of the 5-HTTLPR genotype and unipolar depression: a meta-analysis. Psychological Medicine.

