# Supplementary material for: Meta-analysis of the interaction between serotonin transporter promoter variant, stress, and posttraumatic stress disorder
Source: Sci Rep. 2017 Nov 28;7:16532. doi: 10.1038/s41598-017-15168-0 (PMC5705670; doi:10.1038/s41598-017-15168-0)
Supplement: Supplementary file 1 — Supplementary Materials [file 41598_2017_15168_MOESM1_ESM.pdf]

Title page

Meta-analysis of the interaction between serotonin transporter promoter variant, stress, and posttraumatic stress disorder

Mingzhe Zhao[1,2], Jiarun Yang[1,2], Wenbo Wang[1], Jingsong Ma[1], Jian Zhang[1], Xueyan Zhao[1], Xiaohui Qiu[1], Xiuxian Yang[1], Zhengxue Qiao[1], Xuejia Song[1], Lin Wang[1], Shixiang Jiang[1], Erying Zhao[1], Yanjie Yang[1,*]

[1] No. 157, Baojian Road, Nangang District, Harbin 150081, China.

Tel: +86 451 87502859;

[2] These authors contributed equally to this work.

*Correspondence: Professor Yanjie Yang, Psychology Department of the Public Health Institute of Harbin Medical University, Heilongjiang Province, China.

Address: No. 157, Baojian Road, Nangang District, Harbin 150081, China. Tel: +86 451 87502859; Fax: +86 451 87502885

E-mail: yanjie1965@163.com

Mingzhe Zhao	E-mail: 1018625419@qq.com	Xiuxian Yang	E-mail: 125020874@qq.com
Jiarun Yang	E-mail: 1287991708@qq.com	Zhengxue Qiao	E-mail: 83571560@qq.com
Wenbo Wang	E-mail: 1356517578@qq.com	Xuejia Song	E-mail: 4874452@qq.com
Jingsong Ma	E-mail: 13945105753@126.com	Lin Wang	E-mail: 466373279@qq.com
Jian Zhang	E-mail: tengteng198312@126.com	Shixiang Jiang	E-mail: 32871873@qq.com
Xueyan Zhao	E-mail: 1375970357@qq.com	Shixiang Jiang	E-mail: 32871873@qq.com
Xiaohui Qiu	E-mail: qiuxiaohui@foxmail.com		

Supplementary Table S1. Studies included in the meta-analysis of the interaction between 5-HTTLPR polymorphism, life stress, and PTSD

**Study** / **Items (+/-)**	**Objectives and hypothesis clearly stated**	**Clear eligibility criteria for study participants**	**Clear definition of all variables**	**Statistical methods replicable**	**Assessment of HWE**	**Assessment of ethnicity**	**Mixed ethnicities addressed statistically**	**Sufficient descriptive data (e.g., age, sex, etc.)**	**Genotype frequencies stated**	**Sample in HWE**	**Population stratification**
Kilpatrick et al., 2007	+	–	+	+	–	+	+	+	+	–	+
Grabe et al., 2009	+	–	+	+	+	+	Unnecessary	+	+	+	-
Xie et al., 2009	+	+	+	+	–	+	Unnecessary	+	+	–	Unnecessary
Kolassa et al., 2009	–	–	+	+	+	+	Unnecessary	+	+	+	Unnecessary
Holman et al., 2011	+	+	+	+	+	+	Unnecessary	+	+	+	Unnecessary
Mercer et al., 2012	+	–	+	+	+	+	+	+	+	+	+
Xie et al., 2012	+	+	+	+	–	+	Unnecessary	+	+	–	Unnecessary
Wald et al., 2013	+	–	+	+	–	+	+	+	+	–	+
Pietrzak et al., 2013	+	+	+	+	+	+	–	+	+	+	+
La Greca et al., 2013	+	–	+	+	–	+	–	+	+	+	–
Walsh et al., 2014	+	–	+	+	+	–	–	+	+	+	-
Telch et al., 2015	+	+	+	+	–	+	+	+	+	–	+
Tian et al., 2015	+	–	+	+	–	–	Unnecessary	+	+	–	Unnecessary
Drevo et al., 2016	+	+	+	+	–	–	–	+	+	–	–

Abbreviations: HWE, Hardy–Weinberg equilibrium; 5-HTTLPR, serotonin transporter promoter variant.

Supplementary Table S2. The demographic information for Case group and Control group of studies on the interaction between 5-HTTLPR, life stress, and PTSD included in the meta-analysis.

study	Case group			Control group		
	No. of participants	Female, N(%)	Age, mean(years)	No. of participants	Female, N(%)	Age, mean(years)
Kilpatrick et al., 2007	19	-	-	537	-	-
Grabe et al., 2009	67	64.2	57.9	1596	49.3	57.6
Xie et al., 2009	229	41.9	39.3	1023	54.7	38.9
Kolassa et al., 2009	331	-	-	77	-	-
Holman et al., 2011	303	52.4	51.77	468	-	-
Mercer et al., 2012	66	100	20.3	138	100	20
Xie et al., 2012	719	-	-	5711	-	-
Wald et al., 2013	-	-	-	-	-	-
Pietrzak et al., 2013	13	-	-	136	-	-
La Greca et al., 2013	-	-	-	-	-	-
Walsh et al., 2014	205	-	-	477	-	-
Telch et al., 2015	-	-	-	-	-	-
Tian et al., 2015	64	-	-	119	-	-
Drevo et al., 2016	-	-	-	-	-	-

-, no report in the study.
